# Long-Read Sequencing and Comparative Genome Analyses of Seven *Emergomyces* Type-Strains

**DOI:** 10.1007/s11046-026-01085-2

**Published:** 2026-06-12

**Authors:** Ilan S. Schwartz, Bert Gerrits van den Ende, Ferry Hagen

**Affiliations:** 1 Division of Infectious Diseases, Department of Medicine, Duke University School of Medicine, Durham, NC 27710 USA; 2 Department of Medical Mycology, Westerdijk Fungal Biodiversity Institute, 3584CT Uppsalalaan 8, Utrecht, The Netherlands; 3 Department of Medical Microbiology, University Medical Center Utrecht, Heidelberglaan 100, 3584CX Utrecht, The Netherlands; 4 Institute for Biodiversity and Ecosystem Dynamics, University of Amsterdam, Amsterdam, The Netherlands

**Keywords:** *Emergomyces*, Emergomycosis, Comparative genomics, Nanopore sequencing, Average nucleotide identity

## Abstract

*Emergomyces* (*Ajellomycetaceae*, *Onygenales*) is a genus of dimorphic fungal pathogens that cause severe, opportunistic infections around the world. We used long-read nanopore sequencing to generate de novo genome assemblies followed by comparative analyses for the type-strains of *Emergomyces africanus, Emergomyces canadensis, Emergomyces crescens, Emergomyces europaeus, Emergomyces orientalis*, *Emergomyces pasteurianus*, and *Emergomyces soli*. The average *Emergomyces* genome was found to be 32,965,087 bp in size (range 28,687,700–35,863,369 bp) with GC%-contents of 42.01–45.72%. Average Nucleotide Identity analysis of the seven type-strain genomes with the publicly available *Emergomyces* and *Blastomyces* genomes was performed, showing values of 85–100% similarity between genomes and 85–91% between the twelve species tested. Furthermore, we formally validate the species descriptions for *Emergomyces crescens* and *Emergomyces soli*.

*Emergomyces* is a genus of thermally dimorphic human fungal pathogens that belongs to the order *Onygenales* (family *Ajellomycetaceae*) alongside better-known taxa of human and animal pathogens including *Blastomyces*, *Coccidioides*, *Histoplasma*, and *Paracoccidioides* [1]. *Emergomyces* species have emerged as causes of clinical disease, primarily in immunocompromised patients, around the world [1]. The genus was proposed less than a decade ago to accommodate several pathogens isolated from immunocompromised patients with systemic infections that resembled *Emmonsia parva* (since reclassified as *Blastomyces parvus*) in the mould phase, but which transformed to small yeast-like cells in host tissues, rather than the swollen, sessile “adiaspores” of *E. parva*. Specifically*,* the genus initially included *Emmonsia pasteuriana* (= *Emergomyces pasteurianus*), *Emmonsia orientalis* (= *Emergomyces orientalis*) and *Emergomyces africanus* [1, 2], and were joined soon thereafter by *Emergomyces canadensis* and *Emergomyces europaeus* [3–6]. Lastly, Jiang and colleagues transferred to the genus two non-pathogenic species, *Emmonsia soli* and *Emmonsia crescens,* as *Emergomyces soli* and *Emergomyces crescens*, respectively, based on genetic relatedness [7]. These taxa are outliers in that *E. soli* is a non-pathogen that has been isolated only from soil, and *E. crescens* transforms to swollen adiaspores rather than yeasts, and causes a localized pulmonary disease known as adiaspiromycosis in small mammals [7]. While the geographical distribution of *Emergomyces* appears to be widespread, emergomycosis remains a relatively rare disease, except for *E. africanus* which is among the most common clinically encountered dimorphic fungal pathogens in South Africa, where it causes severe, disseminated infections in persons with advanced HIV disease [8, 9].

With the increasing application of molecular diagnostics to detect and identify fungal pathogens directly from clinical material there is a need for reference genomes. Especially with the rapidly developing applications of metabarcoding and metagenomics in clinical diagnostics [10, 11]. At present, few highly fragmented genomes of *Emergomyces* species have been made publicly available via NCBI Genome. Here, we aim to provide high-quality reference genomes for the type-strains of all seven *Emergomyces* species.

The type-strains of *E. africanus* (CBS 136260^T^), *E. canadensis* (CBS 139872^T^), *E. crescens* (CBS 177.60^T^), *E. europaeus* (CBS 102456^T^), *E. orientalis* (CBS 124587^T^), *E. pasteurianus* (CBS 101426^T^), and *E. soli* (CBS 142607^T^) were obtained from the CBS Culture Collection (hosted at the Westerdijk Fungal Biodiversity Institute, Utrecht, Netherlands). The strains were sub-cultured on Malt-Extract agar (MEA; Oxoid, Basingstoke, United Kingdom) and incubated at 25 °C for 2 weeks to obtain enough biomass for genomic DNA purification using the manual cetyltrimethylammonium bromide extraction protocol [12]. Genomic DNA quality and quantity was assessed by Qubit Broad Range kit (ThermoFisher, Waltham, MA, U.S.A.), 2% agarose gel electrophoresis and by TapeStation using the Genomic DNA Screentape (Agilent, Santa Clara, CA, U.S.A.).

One microgram of genomic DNA was used as input for long-read nanopore sequencing for which the library preparation was performed with the SQK-NBD114.24 kit that enables multiplex sequencing by ligation of unique barcodes per sample (Oxford Nanopore Technologies, ONT; Oxford, United Kingdom). The library was loaded onto R10.4.1 flow cells and raw sequence data was generated with the MinION Mk1D platform (ONT). Raw sequence data was basecalled using the super-accurate mode with dorado v7.8.3 model dna_r10.4.1_e8.2_400bps_sup@v4.3.0 implemented in the MinKNOW v25.03.9 standalone software (ONT). Ultimately, all sequence data per strain was collected into a single FASTQ file and subsequently quality-controlled prior further downstream analysis. For this, FASTQ files were filtered using chopper v0.8.0 for reads of ≥ 1100 bp and a Q-value of ≥ 10, followed by 50 bp removal of the 5′- and 3′-ends of each read [13]. NanoComp v1.12.0 was used to create filtered sequence statistics as listed in Table 1 [13].

**Table 1:** Genome assembly information of the seven sequenced *Emergomyces* type-strains

General summary	*E. africanus* CBS 136260^ T^	*E. canadensis* CBS139872^T^	*E. crescens* CBS 177.60^ T^	*E. europaeus* CBS 102456^ T^	*E. orientalis* CBS 124587^ T^	*E. pasteurianus* CBS 101426^ T^	*E. soli* CBS 142607^ T^
*General sequence statistics*							
Mean read length	3497.6	7002.9	4118.7	4079.1	3176.8	6616.8	5213.6
Mean read quality	20.1	18.9	18.8	18.5	20.3	18.6	19.1
Median read length	2093	4100	2349	2040	2010	3523	3382
Median read quality	23.0	21.0	20.8	20.3	23.2	20.6	21.3
Number of reads	703,855	107,200	242,230	389,145	464,413	173,376	254,962
Read length N50	5139	12,614	6756	7601	4234	12,736	8200
S.D. read length	3735.6	7326.0	4496.5	5214.9	3319.2	7150.5	5046.8
Total bases	2,461,785,846	750,711,355	997,675,177	1,587,347,577	1,475,361,397	1,147,197,235	1,329,266,616
Reads with quality > Q10	703,693 (100.0%) 2461.5Mbp	107,183 (100.0%) 750.7Mbp	242,192 (100.0%) 997.6Mbp	389,088 (100.0%) 1587.3Mbp	464,336 (100.0%) 1475.2Mbp	173,362 (100.0%) 1147.2Mbp	254,938 (100.0%) 1329.2Mbp
Reads with quality > Q15	660,833 (93.9%) 2312.8Mbp	98,452 (91.8%) 689.6Mbp	221,846 (91.6%) 915.2Mbp	353,263 (90.8%) 1447.7Mbp	436,857 (94.1%) 1389.5Mbp	157,749 (91.0%) 1047.9Mbp	235,228 (92.3%) 1220.8Mbp
Reads with quality > Q20	525,407 (74.6%) 1839.9Mbp	66,491 (62.0%) 472.9Mbp	142,520 (58.8%) 604.3Mbp	209,207 (53.8%) 899.1Mbp	351,961 (75.8%) 1123.6Mbp	98,203 (56.6%) 674.7Mbp	166,683 (65.4%) 876.4Mbp
Reads with quality > Q25	218,803 (31.1%) 703.8Mbp	8219 (7.7%) 30.4Mbp	20,072 (8.3%) 51.5Mbp	24,514 (6.3%) 58.4Mbp	152,881 (32.9%) 463.2Mbp	11,270 (6.5%) 39.1Mbp	25,081 (9.8%) 84.5Mbp
Reads with quality > Q30	35,880 (5.1%) 69.1Mbp	543 (0.5%) 0.9Mbp	1386 (0.6%) 2.1Mbp	1555 (0.4%) 2.2Mbp	23,006 (5.0%) 44.0Mbp	692 (0.4%) 1.1Mbp	1601 (0.6%) 2.8Mbp
*Genome statistics*							
Total genome size	32,435,257 bp	33,509,182 bp	32,560,084 bp	35,863,369 bp	34,295,918 bp	33,404,102 bp	28,687,700 bp
GC%	42.01%	44.69%	44.46%	42.27%	45.72%	43.91%	45.46%
Fragments	9	13	22	10	42	9	12
Fragments N50	4,239,726 bp	3,738,050 bp	4,473,560 bp	5,096,094 bp	1,452,906 bp	4,469,582 bp	3,738,837 bp
Largest fragment	9,500,345 bp	5,418,418 bp	7,332,920 bp	6,469,515 bp	4,517,116 bp	9,833,851 bp	6,844,355 bp
Mean coverage	76X	21X	30X	43X	22X	32X	45X
Mitochondrial genome size	22,061 bp	33,725 bp	31,265 bp	42,028 bp	30,657 bp	27,220 bp	24,969 bp
Mitochondrial genome coverage	5117X	1045X	978X	876X	1264X	1197X	1341X
Predicted genes	8390	9763	9788	9792	10,899	9421	8716
*BUSCO analysis using the onygenales_odb10 dataset (4,862 BUSCOs included)*							
Single BUSCOs	4,847 (99.69%)	4,857 (99.90%)	4,852 (99.79%)	4,851 (99.77%)	4,846 (99.67%)	4,850 (99.75%)	4,850 (99.75%)
Duplicated BUSCOs	3 (0.06%)	4 (0.08%)	2 (0.04%)	2 (0.04%)	10 (0.21%)	4 (0.08%)	5 (0.10%)
Fragmented BUSCOs	3 (0.06%)	0 (0.00%)	1 (0.02%)	1 (0.02%)	3 (0.06%)	0 (0.00%)	0 (0.00%)
Missing BUSCOs	9 (0.19%)	1 (0.02%)	7 (0.14%)	8 (0.16%)	3 (0.06%)	8 (0.16%)	7 (0.14%)
*Data availability *via* NCBI BioProject PRJNA1285980*							
NCBI BioSample	SAMN50881230	SAMN49785778	SAMN49785779	SAMN49785776	SAMN50881229	SAMN49785780	SAMN49785777
NCBI Sequence Read Archive	SRR35194778	SRR34394616	SRR34394615	SRR34394618	SRR35194779	SRR34394614	SRR34394617
NCBI Genome	JBRATR000000000	JBVPGW000000000	JBRATP000000000	JBVPGX000000000	JBTGNQ000000000	JBRATQ000000000	JBVPGV000000000

De novo genome assemblies were generated using Flye v2.9.5-b1801 followed by manual curation, which includes (i) removal of short contigs that have low coverage compared to the nuclear content (< 10kbp) are caused by barcode-leakage which is a known phenomenon with the library kit used; ii) checking the circularity of mitochondrial genomes as this software package usually places multiple copies of circular fragments in tandem [14]. The average *Emergomyces* genome was found to be 32,965,087 bp in size with a range of 28,687,700 bp (*E. soli* genome) to 35,863,369 bp (*E. europaeus* genome). The assembled genome sequences in this study of *E. africanus* CBS 136260^T^ and *E. pasteurianus* CBS 101426^T^ consisted each of 9 fragments (Table 1). This is a significant improvement as for both type-strains the genome sequences have been deposited a decade ago in NCBI Genome (GCA_001660665.1 and GCA_001883825.1, respectively) which are highly fragmented with 4444 and 1643 scaffolds, respectively. The genome sequences of *E. europaeus* CBS 102456^T^, *E. soli* CBS 142607^T^, and *E. canadensis* CBS 139872^T^ were assembled into slightly more fragments, being 10, 12 and 13 fragments, respectively (Table 1). The genome sequence of *E. crescens* CBS 177.60^T^ consists of 22 fragments, from the same strain a decade old NCBI deposited genome sequence (GCA_001008285.1) is available that consists of 1734 scaffolds, while that of another strain (UAMH 4076; GCA_002572855.1) is also highly fragmented with 1288 scaffolds. The genome sequence of *E. orientalis* CBS 124587^T^ was the most fragmented with 42 fragments (Table 1). However, this long-read nanopore based assembled genome sequence is an improvement compared to the two publicly available *E. orientalis* genomes deposited in NCBI (GCA_002110485.1 and GCA_020085055.1) which were assembled with 108 and 2,498 scaffolds, respectively. The most accurate number of chromosomes has only been determined for *Histoplasma capsulatum* and *Paracoccidioides brasiliensis* using various karyotyping tools, which demonstrated 5–7 chromosomes for *H. capsulatum* and 4–5 chromosomes in *P. brasiliensis* [15, 16]. There are no karyotyping data available for *Blastomyces* and *Emergomyces* species. To investigate the level of genome completeness we used TeloVision v0.3.2 to detect telomeric regions in the *Emergomyces* genome assemblies [17]. This revealed that the genome of *E. pasteurianus* was nearly fully telomere-to-telomere assembled, as of the 8 nuclear fragments 4 had telomeric regions at both ends. This suggests that *E. pasteurianus* CBS 101426^T^ has 6 chromosomes (Supplementary Data). Two fragments in the genomes of *E. africanus* CBS 136260^T^, *E. europaeus* CBS 102456^T^ and *E. canadensis* CBS 139872^T^ had telomeric regions at both ends, while this was only seen for one fragment in the genomes of *E. crescens* CBS 177.60^T^ and *E. soli* CBS 142607^T^, and no telomere-to-telomere assembled fragments in *E. orientalis* CBS 124587^T^ (Supplementary Data). Given this analysis it is expected that, like other closely related genera, *Emergomyces* species have 5–7 chromosomes. Genome completeness was investigated using compleasm v0.2.7 that assesses the genome sequence against a benchmarking dataset of lineage-specific single-copy orthologs, in this case the onygenales_odb10 dataset [18, 19]. All 7 genome sequences were found to be highly complete, with 4846–4857 BUSCO’s present among the 4862 BUSCO assessed (Table 1). Subsequently, ab initio gene prediction was performed using the Helixer v0.3.4 webtool [20]. The range of predicted genes was found to be 8390–10,899, with the highest number of predicted genes for *E. orientalis* CBS 124587^T^ (Table 1).

The GC% was assessed using the EZBioCloud webtool [21] resulting in values between 42.01–45.72%, which is closely to previously reported GC% values of other *Onygenales* species. Earlier performed DNA-DNA hybridization experiments showed that *E. crescens* CBS 177.60^T^ had a nuclear DNA GC% of 46.8%, and *E. pasteurianus* CBS 101426^T^ (cited as IP 2310.95) a nuclear DNA GC% of 47.3% [22]. The same study reported overall nuclear DNA GC% of 46.1–47.3% for species belonging to other *Onygenales* genera. As the in silico GC% calculation is more accurate than the DNA-DNA hybridization method, this explains the overestimation of GC% for the latter approach.

Finally, we compared the nanopore long-read based genome sequences of the 7 *Emergomyces* type-strains to publicly available genome sequences available from NCBI Genomes. For this, we applied an Average Nucleotide Identity (ANI) calculation using pyani v0.2.13.1 which determines the percentage similarity between genomes by arbitrarily generating *k*-mer fragments (usually 1024 bp) and compare them all against all for every pair of genomes [23]. Other publicly available *Emergomyces* and *Blastomyces* genome sequences at NCBI Genomes were included in this analysis, the strain details and genomic data are presented in Fig. 1 [1, 24, 25]. The two *E. pasteurianus* strains (CBS 101426^T^ and UAMH 9510) had an ANI-value of 100%, this also applies to the two genome sequences of *E. africanus* CBS 136260^T^ (Fig. 1) and to that of both *E. orientalis* strains (5z489 and CBS 124587^T^). This could be expected for these three species as the genome pairs per species are the same strain that has been deposited in different culture collections. However, given the differences in used next-generation sequencing technology (viz. short-read *versus* long-read), different assemblers used, and the large difference in fragments per genome it was unexpected that the ANI-values were in all three cases 100%. For example, two different strains of *E. crescens* (CBS 177.60^T^ and UAMH 4076) have an ANI-value of 93%. All other *Emergomyces* genome sequences have ANI-values of 85–89% when compared to any of the other genome sequences in this analysis, even those from *Blastomyces* species and vice versa. This percentage is more in the expected range for different species.

**Fig. 1:**
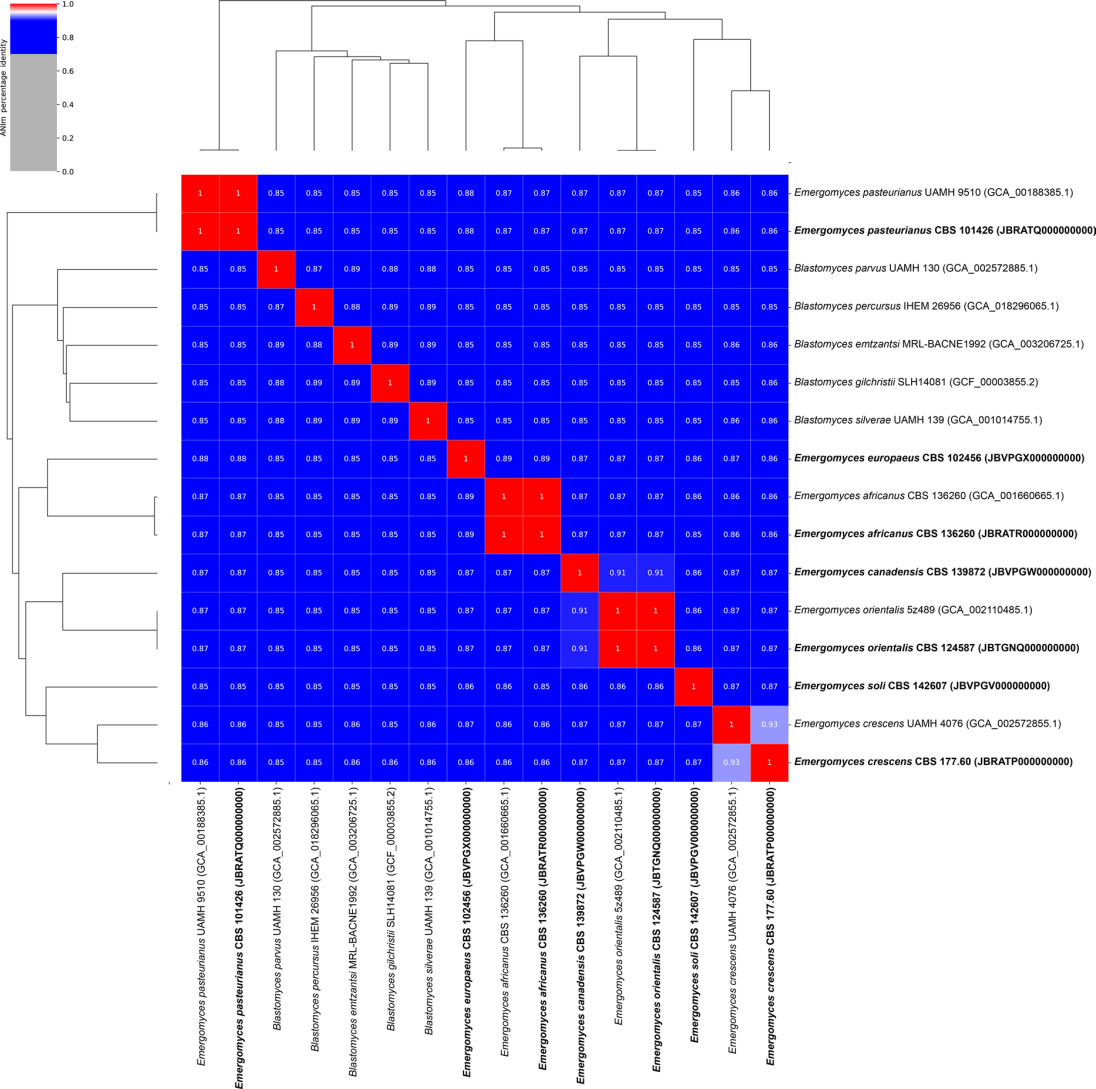
Average Nucleotide Identity (ANI) analysis using pyani v0.2.13.1 which determines the percentage similarity between every pair of genomes. [20] Other publicly available *Emergomyces* and *Blastomyces* genome sequences from NCBI Genomes were included in this analysis. Species names are followed by the (type-)strain accession number and NCBI Genomes accession number (between brackets). Information presented in bold identifies the here described and analysed de novo genome assemblies. Raw sequence reads and de novo genome assemblies are available via the NCBI BioProject database under accession number PRJNA1285980. Additionally, the de novo genome assemblies can be accessed via the DANS-KNAW Data Station Life Sciences (https://doi.org/10.17026/LS/SXUBNO)

Lastly, we observed that the descriptions of the new combinations for *E. crescens* and *E. soli* were in 2019 not validly described by Jiang and colleagues [7]. Hence, we here take the opportunity to formally validating these names:

*Emergomyces crescens* (C.W. Emmons & Jellison) Y.P. Jiang & de Hoog, comb. nov.—Mycobank MB862275.

Basionym: *Emmonsia crescens* C.W. Emmons & Jellison, Ann. New York Acad. Sci. 89: 98, 1960.

Synonyms: *Chrysosporium parvum* var. *crescens* (C.W. Emmons & Jellison) J.W. Carmich., Canad. J. Bot. 40: 1164, 1962.

*Emmonsia parva* var. *crescens* (C.W. Emmons & Jellison) Oorschot, Stud. Mycol. 20: 59, 1980.

*Emergomyces crescens* (C.W. Emmons & Jellison) Y.P. Jiang & de Hoog, Mycopathologia 185: 626, 2020, nom. inval.

*Ajellomyces crescens* Sigler, J. Med. Vet. Mycol. 34: 305, 1996.

*Emergomyces soli* (Y.P. Jiang, Borman & de Hoog) Y.P. Jiang & de Hoog, comb. nov.—MycoBank MB862276.

Basionym: *Emmonsia soli *Y.P. Jiang, Borman & de Hoog [as ‘*sola*’], Fungal Diversity 90: 262, 2018.

Synonym: *Emergomyces soli* (Y.P. Jiang, Borman & de Hoog) Y.P. Jiang & de Hoog, Mycopathologia 185: 626, 2020, nom. inval.

## Data Availability

*Emergomyces* type-strains are available via the CBS Culture Collection (Westerdijk Institute, Utrecht, The Netherlands). The basecalled and quality controlled FASTQ reads and the genome sequences are available via the NCBI BioProject database under accession number PRJNA1285980. Additionally, the de novo genome assemblies can be accessed via the DANS-KNAW Data Station Life Sciences (https:/doi.org/10.17026/LS/SXUBNO).
